# Evidence of copper (nano)formulation biotransformations triggered by *Botrytis cinerea* on grapevine leaves for targeted plant protection

**DOI:** 10.1039/d5en01102g

**Published:** 2026-02-24

**Authors:** Diana Salvador, Sónia Rodrigues, Artur Alves, Cátia Fidalgo, Sandra Rodrigues, Hiram Castillo-Michel, Astrid Avellan

**Affiliations:** a Department of Biology and Centre for Environmental and Marine Studies (CESAM), Universidade de Aveiro Portugal diana.salvador@ua.pt; b Department of Environment and Planning and Centre for Environmental and Marine Studies (CESAM), Universidade de Aveiro Portugal; c European Radiation Synchrotron Facilities, Grenoble France; d Géosciences Environnement Toulouse (GET), CNRS, Univeristé de Toulouse France astrid.avellan@cnrs.fr

## Abstract

The present work aimed at unravelling if fungal inoculation on grapevine leaves could trigger the dissolution of foliarly deposited Cu (nano)formulations, and how this would impact Cu translocation *in planta*. Leaves of grapevine seedlings were exposed to 3.3 μg of Cu (5 μg cm^−2^). Formulations of contrasting solubilities (micro-sized conventional Bordeaux mixture, CuO-nanoparticles (CuO-NPs), and CuSO_4_) were applied to grapevine leaves, followed by the inoculation of *Botrytis cinerea* spores on top of the deposited Cu formulations. Nine days after Cu deposition, and six days post inoculation, Cu distribution and transformations were assessed at the leaf surface using micro-X-ray fluorescence and X-ray absorption near-edge structure spectroscopy. Cu was also quantified in non-exposed tissues to evaluate the role of fungal-triggered transformations on Cu translocation. For all non-inoculated formulations, Cu remained largely untransformed at the leaf surface. After inoculation of *B. cinerea*, Cu was partly found complexed with carboxylate- and thiol-containing compounds, associated with partial Cu reduction, with similar patterns across all (nano)formulations. This was mainly attributed to the presence of fungal metabolites. Despite these transformations, Cu did not significantly translocate *in planta*, with all the taken-up Cu found on and/or within exposed leaves. This work suggests that these approaches could lead to more efficient plant protection strategies by (i) increasing leaf affinity of Cu-based compounds, while (ii) triggering ionic Cu release thanks to a pathogen-triggered dissolution.

## Abstract 2

The present work aimed at unravelling if fungal inoculation on grapevine leaves could trigger the dissolution of foliarly deposited Cu (nano)formulations, and how this would impact Cu translocation *in planta*.

## Graphical abstract

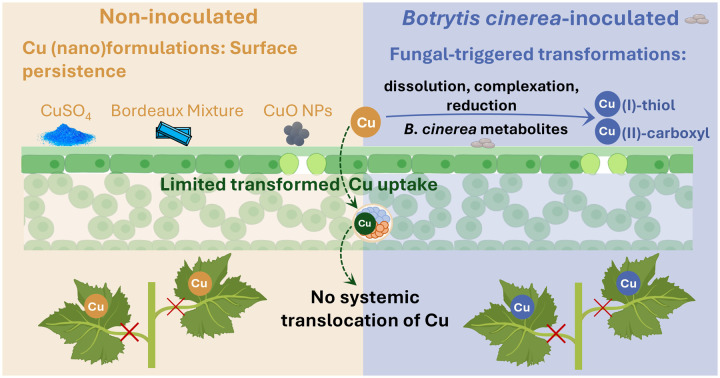

**Environmental significance**

Understanding how pathogen presence and associated metabolites modulate the behavior of Cu-based (nano)formulations on/in plant leaves is essential to optimize their effectiveness and environmental impacts. Cu (nano)formulations with contrasting solubilities (CuSO_4_, Bordeaux mixture, CuO-NPs) and presenting different leaf affinities (CuSO_4_ < Bordeaux mixture < CuO-NPs), were all persisting untransformed on leaves. When inoculated with *Botrytis cinerea* spores, they all dissolved and chelated to carboxylate- and thiol-groups present in biomolecules. Despite these transformations, Cu translocation to other plant tissues was not promoted, and no persistence of nanoforms was found *in planta*. These results demonstrate that Cu nanoformulations can efficiently target plant leaves while releasing ionic Cu upon pathogen-trigger. These findings may be helpful for designing safer and efficient nano-based fungicides for the near future.

## Introduction

The pressure on copper (Cu) usage in agriculture is rising due to (i) the increase of organic farming in Europe,^1,2^ (ii) climatic changes favoring fungal diseases outbreaks,^3–5^ and (iii) current restriction in the European Union (European Commission Implementing Regulation 2018/1981) for the allowed application dosages, namely 4 kg per hectare per year (40 μg cm^−2^ per year).^3^ These regulation changes have led to the application of Cu-based fungicides in a dose-efficient way to ensure food security while meeting an increase in world population demands.^6,7^ However, to better achieve this dose-efficiency, there is a need to understand how Cu-based fungicides act against leaf pathogens.

The presence of ionic Cu in is crucial for fungicide effect. The redox cycle between Cu^+^ and Cu^2+^ in the Fenton- and Haber–Weiss-like Cu reaction,^8,9^ generates ROS, including hydroxyl radicals. This causes pathogen toxicity by directly acting on lipids, proteins, and nucleic acids,^10,11^ leading to cell death. Cu^2+^can also disrupt cell membranes and walls by binding to their negatively charged groups, resulting in increased membrane permeability and leakage of cellular content due to burst release.^10–12^ Therefore, to effectively protect plants from foliar pathogens, Cu-based fungicides need to remain on the surface of the leaf while timely delivering ionic Cu upon infection.

Several strategies allow foliar delivery of Cu^2+^, including: (i) Cu salts (*e.g.* CuSO_4_), that provide Cu^2+^ at the moment of application but generate plant stress, leaf phytotoxicity,^13,14^ and present low leaf adhesion,^15^ (ii) precipitated-based Cu fungicides, as Bordeaux mixture, a mix of CuSO_4_ and Ca(OH)_2_, that presents decreased phytotoxicity^16^ but has low leaf affinity, and (iii) nano-based strategies, as CuO-nanoparticles (CuO-NPs), that present 5× higher leaf retention and lower leaf toxicity than CuSO_4_.^15^

The higher leaf adhesion presented by CuO-NPs^15,17^ allows to apply the formulation at the intended targeted tissue,^15,18,19^ without the need to reapply following each rain event due to decreased wash-off.^3,17,20,21^ Consequently, the application frequency and intensity are decreased, bringing environmental benefits and making CuO-NPs an interesting cost-effective alternative. Aside from the increased leaf affinity, Cu-based nanoformulations have been proposed as an effective plant protection,^21–29^ showing potential as fungicides with decreased Cu leaching rate, even in greenhouse conditions,^23^ and with targeted-release directed not only to the plant tissue but also to inner leaf compartments.^21^ Nonetheless, the link between the release of Cu^2+^ at the leaf interface in the presence of pathogenic fungi, the associated Cu transformations on/in the leaf, and the possible translocation to other plant tissues remain overlooked.

Addressing the effect of the pathogen on plant-applied Cu (nano)formulations is of utmost importance, especially considering that biomacromolecules released by pathogens may interact with the Cu (nano)formulations and modulate their reactivity. For instance, pathogen-mediated transformation of CuO-NPs was previously reported for the fungus *Botrytis cinerea* in *in vitro* experiments.^30^ This study showed that *B. cinerea* can transform CuO-NPs into a chelated form of Cu, namely associated with oxalate and catechol-siderophore types, when these are applied at 0.8 mg cm^−2^.^30^ It is worth noting that this Cu concentration is above the current EU limitation, namely 0.04 mg cm^−2^, and it may not represent a realistic, *in planta* scenario. It remains unclear if this type of biotransformation can take place *in planta* with a Cu dose within the legislation restrictions upon *B. cinerea* inoculation.

*B. cinerea* is a well-known plant pathogen, with a well described metabolome, and causal agent of grey mold in numerous crops,^31^ responsible for yield and production losses,^32^ thus being one of the most important grapevine pathogens.^33^ Upon disease outbreak, *B. cinerea* produces numerous metabolites, namely organic acids, enzymes and proteins, to facilitate plant-tissue penetration. To enhance its colonization, *B. cinerea* produces organic acids, namely oxalic acid, to acidify the surrounding media,^34–36^ and/or to suppress plant defenses.^32,37^ It can be hypothesized that the presence of *B. cinerea* following the application of CuO-NPs (representing a preventive protection strategy) may trigger the release of Cu^2+^,^30,38^ since lower pH values could induce their dissolution,^39^ releasing ionic Cu, the major fungicide form of Cu. However, at the same time, the metabolites produced by the fungus while growing may also chelate the released Cu^2+^, which in turn could impair the reactivity of the Cu formulation applied.^30,40^ Capturing this dynamic interplay is of most importance to evaluate the potential of CuO-NPs' triggered-release as well as the consequences of these transformations on the fate of Cu at the leaf surface and within the plant system.

The present work investigates pathogen-induced transformations on leaf-applied Cu (nano)formulations (5 μg Cu per cm^2^), and whether the resulting transformations may influence the subsequent transport *in planta*. To address this, transformations and fate of different Cu formulations of various solubilities and reactivities, namely, CuO-NPs, Bordeaux mixture and CuSO_4_, were studied with and without the presence of *B. cinerea* in grapevines leaves. Scanning electron microscope (SEM) images were obtained to assess both the formulations and the spores' distribution on the leaf, and Cu distribution and local speciation were assessed, by μ-XRF and XANES, respectively. Moreover, Cu quantification was assessed by ICP-MS to infer about uptake and translocation to other plant tissues. Addressing the biotransformations of Cu (nano)formulations at the leaf surface following fungal inoculation may help to design more dose-effective Cu fungicides, by predicting possible pathogen-driven reactivity while aiming at retention of Cu on the applied tissues.

## Experimental section

### Copper formulations properties and dissolution

In this study three formulations were used: CuSO_4_·5H_2_O, Bordeaux mixture, and CuO-NPs. CuO-NPs and CuSO_4_·5H_2_O, were both purchased from Sigma Aldrich (USA). Concentrated Bordeaux mixture was prepared in the lab according to the literature, in a 10 : 10 : 100 ratio of CuSO_4_·5H_2_O, calcium hydroxide, Ca(OH)_2_, and water.^16^ Cu formulations were further characterized by SEM. For this, leaf disks containing a drop of each Cu treatment at 0.1 g of Cu per L were collected, placed in an aluminum stub with double-sided carbon tape to fixate the sample, followed by carbon deposition with graphite. Samples were then placed in a vacuum chamber overnight and observed in a scanning electron microscope Hitachi SU70, at a resolution of 1.0 nm at 15 kV and working distance (WD) of 4 mm. ImageJ software (version 1.52a)^41^ was used to measure the size of the Bordeaux mixture microparticles (*n* = 60) in the SEM micrographs. Dissolution tests were performed for Bordeaux mixture using the same methodology used in a previous study for Cu-NPs.^15^ Briefly, Bordeaux mixture was prepared at 1 mg L^−1^ and placed under agitation for 7 days. Aliquots were collected at 0, 3 and 7 days. Further details are presented in SI (SI, ‘Methods’ section).

### Seed germination and plant growth

The grapevines used in this study were germinated in laboratory conditions, as follows: grapevine seeds of the variety *Vitis vinifera* subsp. sylvestris were hydrated for 48 h followed by soaking in H_2_O_2_ 0.5 M overnight, washed, and placed in a solution of 2.6 mM of gibberellic acid (GA_3_) overnight.^42^ After 3 weeks of stratification, the seeds were washed with sterilized dH_2_O, scarified and placed in GA_3_ 2.6 mM for 18 h.^43^ Scarified seeds were then placed in Petri dishes, with sterilized and moistened absorbent paper and incubated with a photoperiod (light/dark cycle of 16/8 h) at 25–28 °C until the development of the first two leaves. These seedlings were then transferred to pots with acid washed, sterilized sand, and kept at the same conditions as those used for germination. The protocol used for acidic sand washing is presented in SI, including the Cu concentration measured in the sand. The plants were watered every two days with modified Hoagland solution (containing all macro- and micronutrients except Cu) at 25% strength (Table S1). Plants were grown in sand and watered with modified Hoagland solution, namely without Cu, to limit its supply to the plant system. This way, the only Cu source would be the one applied to the exposed leaves, thus allowing to assess the possible Cu translocation to non-exposed tissues.

### *Botrytis cinerea* growth conditions and spore suspension preparation

*Botrytis cinerea* strain B05.10 was used in this study. The culture was maintained in a modified tomato juice medium, consisting of 50% (v/v) tomato pulp and 2.4% (m/v) agar, at 25 °C in the dark. After 10 days, a well-sporulated culture was used to prepare a spore suspension. Briefly, a part of the mycelium was removed from the plate and submerged in a microcentrifuge tube containing 0.3% NaCl, for spores' release. At this point, it was also considered that some metabolites produced by the fungus *in vitro* could be released to the prepared suspension along with the spores and be transferred to the leaves upon plant inoculation. Spores' concentration was measured using a Neubauer chamber and the final concentration was adjusted to 1 × 10^5^ spores per mL.

### Plant exposure to copper formulations and *B. cinerea* inoculation

Plants (*n* = 40) at the 7th leaf stage were foliar exposed to the Cu formulations, namely: CuO-NPs, CuSO_4_·5H_2_O (ionic control), and Bordeaux mixture (conventional fungicide). Sterile dH_2_O was used as Cu-free control. Two leaves per plant (5th and 6th leaves) were trapped with the abaxial side facing up for drop deposition. Three drops of 40 μL, of each formulation, were added to the leaf at 0.0275 g of Cu per L, giving a surface area concentration of 5 μg of Cu per cm^2^ per leaf (6.6 μg of Cu/plant), 1/8 of the maximum concentration allowed in European agricultural practices in a year (40 μg cm^−2^ per year),^3^ allowing for multiple reapplications while working under the Cu dose restrictions. After exposure, the plants were incubated in a growth chamber with a light/dark cycle of 16/8 h, 25 °C/22 °C and 95% of relative humidity.

Three days after the formulations' deposition, half of the plants were inoculated with a spore suspension of *B. cinerea* (in 0.3% NaCl), while in the other half, the non-inoculated control, 0.3% NaCl was added. Three drops of 40 μL spores' suspension at 1 × 10^5^ mL^−1^ (or 0.3% NaCl) were placed on top of the Cu formulations' drops. To ensure the spores were viable, the same volume of spore suspension was inoculated in a potato dextrose agar (PDA) plate to assess spores' growth and viability. Three sets of plants were prepared: (i) for Cu quantification with ICP-MS (*n* = 24), (ii) for assessing Cu distribution and speciation with micro-X-ray fluorescence (μ-XRF) mapping and X-ray absorption near-edge structure spectroscopy (XANES), respectively, at the European Radiation Synchrotron Facility (ESRF) (*n* = 8) and (iii) for spores' detection with SEM (*n* = 8).

### Plant tissue sampling and copper quantification

After 9 days of Cu exposure and 6 days of *B. cinerea* inoculation, plant tissues were sectioned according to the following: (i) the two exposed leaves, (ii) the tissues between the exposed leaves, composing of the two petioles and the stem between them, (iii) the above tissues, composed of the leaves, petioles, and stem, above the top exposed leaf, (iv) the below tissues, composed of the leaves, petioles, and stem below the bottom exposed leaf, and (v) the roots. Tissues were collected within this period of time to increase the chances of interaction between the Cu formulations and the spores while avoiding the risk of leaf falling. Tissues were ground with liquid nitrogen and dried at 70 °C for 7 days. The dried powder was then acid-digested with a mixture of 0.5 mL of nitric acid and 0.25 mL of hydrogen peroxide. This mixture was left to pre-digest overnight and then placed in a microwave digestor (Speedwave SW4, Berghof, Germany) at 170 °C for 10 minutes following a second temperature cycle at 200 °C for 15 minutes. A second digestion cycle was performed after adding 0.25 mL of HCl. The digested samples were then diluted to a final concentration of 1% (v/v) HNO_3_ for Cu quantification with (ICP-MS) (details below). The initial solutions/suspensions of Cu formulations were also analyzed to assess the initial Cu mass and a proxy of the initially applied Cu mass. The total Cu in plant was calculated as the sum of the Cu quantified in the collected tissues.

### ICP-MS analysis and quality controls

Cu analysis in the extracts was performed by inductively coupled plasma mass spectrometry (ICP-MS) (Agilent 7700 ICP-MS equipped with an octupole reaction system (ORS) collision/reaction cell technology to minimize spectral interferences) using rhodium (103Rh) as internal standard. The calibration was established with a certified reference material (https://www.cpachem.com, traceable to NIST), verified with an independent standard (recoveries between 92 and 104%; average = 98%) and check standards were run every 15 samples (recoveries between 96 and 109%; average = 102%). The quantification limit of the instrument was 0.5 μg L^−1^. Instrumental precision of determinations performed in duplicate was always <5%.

### Leaf tissue sampling for surface mapping and copper speciation assessment

μ-XRF mapping and XANES analysis were performed at the ID-21 beamline in the European Synchrotron Radiation Facilities (ESRF, France), under the proposal number ES-1473 (generated dataset available at https://doi.esrf.fr/10.15151/ESRF-ES-1568128044). Inoculated and non-inoculated leaves, exposed to the different Cu formulations, were collected at the ESRF and leaf disks of 9 mm^2^ were sectioned at the drops' area for surface analysis. The parameters used at the beamline ID-21 mimic the ones used in the previous beamtime at the ESRF.^15^ μ-XRF maps were performed with a ∼0.8 × 0.3 μm beam spot size at 9.1 keV and XANES scans at Cu K-edge (8.98 keV) were performed from 8.95 to 9.2 keV using a 0.5 eV step size and 100 ms dwell time in Cu hotspots/points of interest (POIs), allowing to assess the Cu speciation on specific sample locations. PyMCA software (version 5.9.1) was used to correct for I0 and deadtime, normalize and fit the fluorescence spectra, and to obtain the overlaying distribution maps as RGB images.^44^ XRF fluorescence counts were normalized to be comparable among Cu formulations and DIW control. XANES spectra were evaluated with Orange Data Mining software (version 3.35.0) with the “Spectroscopy” add-on (version 0.8.1).^45^ The second derivative of XANES spectra was calculated by averaging 19 floating points to reduce the noise contribution to the signal, and principal component analysis (PCA) was performed on the intensity of the vector-normalized second derivative at each energy step (0.5 keV). Larch software^46^ (version 0.9.68) was used for spectra normalization and linear combination fitting. XANES standards used were prepared at 1 g of Cu per L. For additional details on the analyzed standards see Table S1 and Fig. S1.

### Detection of *B. cinerea* in the leaf tissues

To confirm the presence of the pathogen and its contact with the Cu formulations, the Cu-exposed and inoculated leaves, as well as the non-inoculated controls, were collected and prepared for SEM, to observe the spores and their germination status. For this, leaf disks of 6 mm containing the exposed and inoculated area (or the non-inoculated control) were excised from the plants at the collection point (9 days after Cu exposure and 6 days post inoculation (dpi)). Each sample was prepared and analyzed following the methodology described above for the SEM analysis.

### Statistical analysis

Statistical analysis was performed using R (version 4.4.3)^47^ and R Studio software (version RStudio 2024.12.1+551). Normality and heteroskedasticity were assessed prior to performing hypothesis tests. Cu quantification was compared across the different Cu formulations, for control and inoculated samples (Kruskal–Wallis followed by pairwise Dunn test with Bonferroni *p*-value correction). The assessment of statistically significant differences between the control and the inoculated plants for each Cu treatment (*t*-test) was also performed.

## Results and discussion

### Copper formulations properties

CuO-NPs were characterized in a previous study, where their particle size, XANES profile, and dissolution rates are reported.^15^ CuO-NPs were 100% CuO, with a nominal size of 54.7 ± 18.3 nm and a zeta potential of −11.8 ± 0.51 mV (in MilliQ water pH 6.8). In the same study, CuSO_4_ speciation was also assessed by XANES.^15^ The XANES spectra of these Cu formulations, along with all the Cu references considered for this study, are shown in Fig. S1.

Cu speciation in Bordeaux mixture was studied by XANES. Results of the PCA on individual spectra and Linear combination fitting (LCF) indicate that the atomic environment of Cu from Bordeaux mixture is linked to hydroxides, although LCF indicates a minor contribution of other Cu-based compounds (Fig. S3). Bordeaux mixture protocols is indeed known to lead to the precipitation of brochantite (Cu_4_(SO_4_)(OH)_6_).^48,49^ Since the main Cu atomic environment in Bordeaux mixture appeared to be linked to a hydroxide group, the Cu(OH)_2_ reference compound was used for the PCA analysis.

Ionic Cu release from the studied formulations was assessed by dissolution tests in DIW. Results indicate that for Bordeaux mixture around 47.4 ± 4.3% of Cu was immediately dissolved in DIW (*t*_0_) and after 7 days, 62.3 ± 10.8% of the Cu from the Bordeaux mixture was in solution (Fig. S4). In similar conditions, 1.9 ± 0.2% of Cu from CuO-NPs were dissolved in DIW on day 0 and after 7 days, 42.8 ± 6.9% of the Cu was in solution (Fig. S4). Overall, Bordeaux mixture and CuO-NPs had contrasting dissolution profiles in DIW, confirming the different Cu^2+^ release rate, a proxy for the formulation reactivity.

**Fig. 1:**
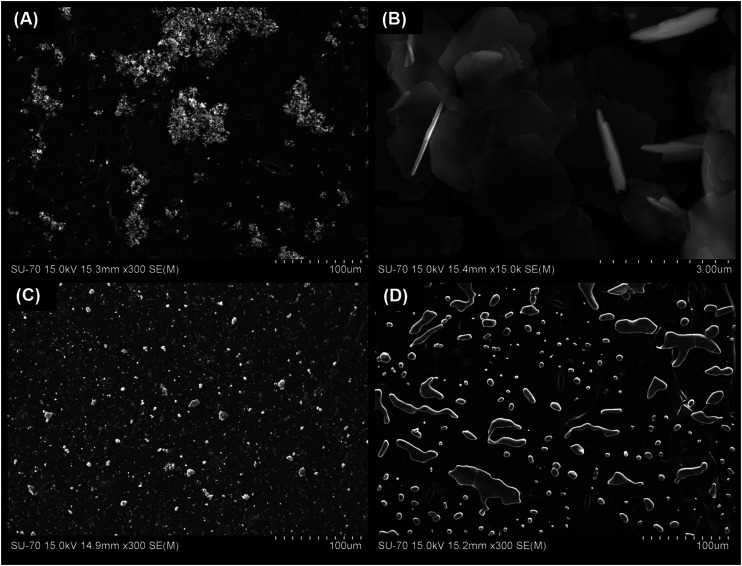
SEM images showing the three Cu formulations at the abaxial surface of the leaf, namely: (A) and (B) Bordeaux mixture, (C) CuO-NPs and (D) CuSO_4_. Focus is given to the microparticles of Bordeaux mixture (B) as there is limited information in the literature regarding the characterization of this formulation.

To complement this characterization, distribution of the Cu formulations following leaf deposition was studied with SEM. SEM micrographs of Bordeaux mixture, CuO-NPs and salt control, CuSO_4_, applied to the leaf surface are shown in Fig. 1. Bordeaux mixture results are presented in Fig. 1A and B, showing precipitates with a size of 2.17 ± 0.47 μm. Although it is expected that Bordeaux mixture will form brochantite crystals in the micro-scale range, the currently available literature did not provide enough information to compare these results. In fact, the literature is scarce regarding the characterization of Bordeaux mixture and to the best of our knowledge, this is the first report of the compound size, Cu speciation and associated dissolution in DIW. CuO-NPs are also dispersed on top of the leaf (Fig. 1C), and similarly to the Bordeaux mixture, there are some surface areas where this nanoformulation appears in aggregates in the micro-scale range (6.43 ± 2.36 μm). Regarding CuSO_4_, SEM observation showed liquescent material at the leaf surfaces (Fig. 1D). This is because CuSO_4_ is highly hygroscopic,^50^ capturing moisture from the air. Following the characterization of the Cu formulations, the leaf distribution was assessed by μ-XRF.

### Mapping the copper distribution in the non-inoculated leaves using μ-XRF

**Fig. 2:**
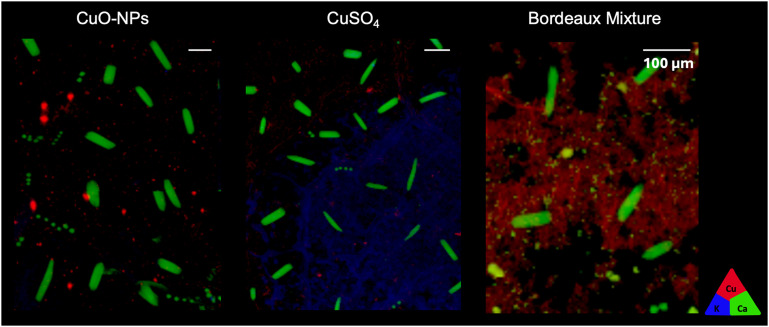
μ-XRF maps showing Cu accumulation in the surface and/or associated with the leaf vasculature, for CuO-NPs, CuSO_4_, and Bordeaux mixture. The triangle represents the color code used: potassium (K Kα) is represented in blue, calcium (Ca Kα) in green and copper (Cu Kα) in red. White bar represents 100 μm.

The Cu distribution profile was assessed by mapping the leaf surface with μ-XRF, 9 days after Cu exposure. At this exposure time, the Cu formulations were detected on the surface of the leaf and/or associated with the vasculature (Fig. 2) of the control samples. In CuSO_4_ and CuO-NPs formulations, Cu was found on the surface and associated with vasculature features (Fig. S5 for vasculature location). In the Bordeaux mixture formulations, no areas containing vasculature tissues were present in the leaf disk analyzed, hence it was not possible to assess the presence of vasculature-associated Cu. It was worth noting that despite these differences in μ-XRF maps, calcium (Ca) hotspots were observed (green, in Fig. 2) in all samples. These Ca crystals likely correspond to calcium-oxalate precipitates, as previously reported in plant leaves, particularly in work assessing plant metal contaminants detoxification.^51^

The μ-XRF maps also allowed to infer about the possibility of Cu uptake after leaf exposure (Fig. 2). Since the Cu signal is recorded among a couple of μm thickness, vasculature-associated Cu could represent Cu localized within the vascular system, and not solely the Cu deposited at the surface of leaf-vasculature tissues. However, it was not possible to address the temporal dynamics of this Cu uptake and/or immobilization since the μ-XRF results here presented are static snapshots collected at the 9-days post exposure rather than time-resolved process. Nonetheless, Cu fluorescence signal intensities in Fig. 2 suggests either (i) that part of the Cu signal could not be seen due to the size of the nano-objects/ionic Cu deposition being smaller than the beam spot and leading to very small concentrations, or (ii) higher Cu uptake rates for CuO-NPs and CuSO_4_ than Bordeaux mixture, since the intensity of fluorescence differs while these μ-XRF maps were obtained on samples of similar thickness and were performed within the drop deposition area. Indeed, Cu uptake was anticipated for both CuSO_4_ and CuO-NPs, as it had been observed in our previous study using a similar setup.^15^ Furthermore, the uptake of elements from nano-based materials with sizes up to 100 nm has been demonstrated in previous studies and experimental systems,^52–55^ supporting the hypothesis of uptake of the 55 nm CuO-NPs used in this study.

Due to the size of the particles, the uptake of Bordeaux mixture is less likely to occur. The Bordeaux mixture prepared and studied in this work had particles of approximately 2 μm, and currently, there is only data regarding foliar uptake (through the apoplast pathway) of particles as large as 200 nm.^56,57^ For uptake to occur in Bordeaux mixture-exposed leaves, the particles would need to undergo dissolution at the leaf surface. According to the dissolution tests, Bordeaux mixture can dissolve in DIW. Another hypothesis for this higher Cu signal observed for the Bordeaux mixture, could be a different accumulation distribution on top of the leaf. It has been reported that NPs tend to aggregate and sediment in the air–liquid interface of the drops while drying, forming a “coffee ring” pattern,^58^ while this has not been reported for salt-based formulations. Overall, it seems that the initial speciation influences the Cu accumulation at the leaf surface and the uptake, since results suggest that uptake of CuO-NPs and CuSO_4_ has likely occurred. To further understand if these distribution patterns and possible uptake are a consequence of leaf surface transformations, Cu speciation changes were assessed by XANES.

### Copper speciation transformations on the surface of non-inoculated leaves

**Fig. 3:**
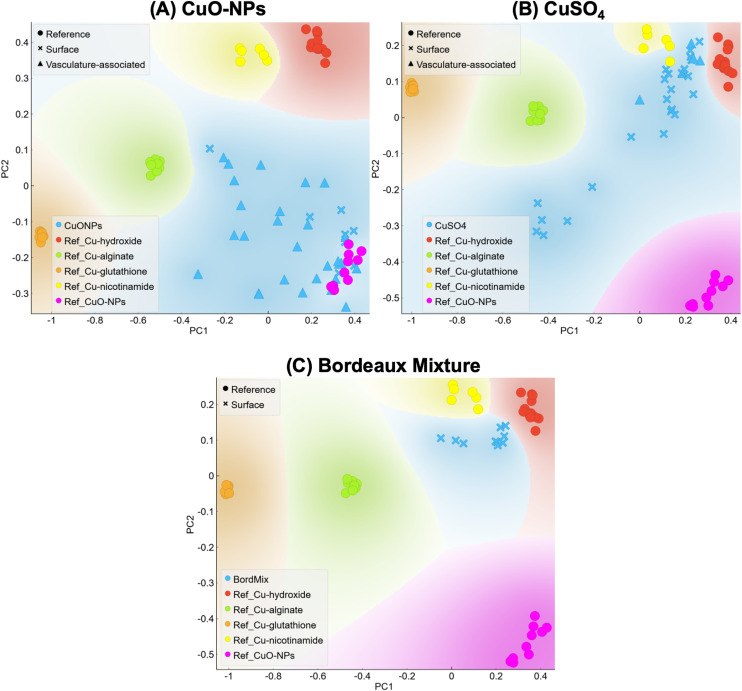
PCA analysis representing XANES results for (A) CuO-NPs, (B) CuSO_4_ and (C) Bordeaux mixture. Samples are compared to the following references: Cu–hydroxide (red), Cu–alginate (Cu–carboxyl, green), Cu–thiol (Cu–glutathione, orange), Cu–nicotinamide (Cu–amine, yellow) and CuO (CuO-NPs, pink). Symbols indicate Cu references, Cu at the leaf surface, and Cu associated with the leaf vasculature.

In leaves exposed to CuO-NPs, Cu detected at the leaf surface was mainly in the CuO-NPs form (Fig. 3A), while Cu associated with the vasculature was found both in the CuO-NPs form and association to some carboxyl groups (Cu–alginate reference). Detection of untransformed CuO-NPs at the surface of the leaf is in accordance with a previous study performed by our group using a similar setup,^15^ where CuO-NPs remained untransformed for up to 25 days of exposure. In the present work, Cu associated with the vasculature was mainly transformed, suggesting, Cu uptake and entrance into the vasculature. The complexation of Cu with carboxyl groups suggests either CuO-NPs dissolution and complexation of the released Cu^2+^ to the carboxyl groups located in cell walls,^59,60^ or biocorona formation implying protein coating associated to/with the CuO-NPs surface.^61^ Overall, results suggest that a fraction of Cu from CuO-NPs was likely taken up and reached the leaf vasculature or its surroundings within the 9 days of exposure.

Cu detected in CuSO_4_-exposed leaves, was not found in the initially applied chemical form, and displayed a Cu–OH type of environment, with some location showing Cu linked to amine and/or carboxyl groups (Fig. 3B). These results indicate that Cu from CuSO_4_ was mainly associated with the hydroxyl groups present on the leaf surface. A fraction of this Cu was associated to amine and carboxyl suggest that some Cu could have been taken up into plant leaves and associated with functional groups there, likely present in metalloproteins and/or cell walls.^59^ Further, a group of points of interest (POIs) was not associated with any of the selected Cu references (Fig. S6). This group presents a speciation similar to some of the Cu associated with the vasculature of CuO-NPs-exposed leaves (Fig. S7), and it can be hypothesized that this Cu from CuSO_4_ may be Cu taken up to inner leaf compartments. The role of the leaf microbiome on the observed transformations for both CuO-NPs- and CuSO_4_-exposed leaves cannot be ruled out. Indeed, previous studies on tobacco plants demonstrated that Cu-based formulations can modulate the phyllosphere.^62^ Therefore, Cu exposure likely triggered changes on the leaf microbiome, consequently causing Cu speciation changes. Lastly, Cu from Bordeaux-mixture exposed leaves were found in the untransformed (Cu–OH local atomic environment) form (Fig. 3C), confirming that this formulation mainly remained untransformed at the leaf surface.

To further assess if the Cu formulations applied to the leaf could suffer additional transformations and/or changes in the spatial distribution once in contact with *B. cinerea*, the inoculated samples were also assessed by μ-XRF and XANES.

### Copper speciation transformations triggered by inoculation of *B. cinerea*

Elemental maps obtained with μ-XRF results showed that, in the tested timeframe, namely 9 days of Cu exposure and 6 dpi, the inoculation of *B. cinerea* had limited impact on the Cu distribution. Cu distribution was similar for both *B. cinerea*-inoculated and control samples (Fig. S8). This suggests that there was no Cu remobilization due to the inoculation of the leaf, as Cu was detected either in the surface or associated with the vasculature, similarly as the non-inoculated samples. Whereas XANES' results showed that the inoculation of *B. cinerea* spores and metabolites triggered Cu speciation transformations.

Although spores were viable, as observed in the plate controls, and that SEM micrographs confirmed their presence on the leaf surface 6 dpi, it should be noted that they were present in limited numbers at the surface (Fig. S9 and S10). This, associated with the lack of disease symptoms suggests that despite the high humidity (95%) and temperature between 22–25 °C, the experimental conditions were not able to trigger the infection. Therefore, it was hypothesized that disease development was likely limited by the absence of foliar lesion. *B. cinerea* usually requires a physical damage in host tissue for colonization^36^ and our setup did not include a leaf lesion, since it could alter the behavior of Cu formulations on the leaf surface, or even promote uptake. Hence, leaves were not considered infected in this work, even though they were inoculated with *B. cinerea* spores. It could also be considered that this situation (presence of *B. cinerea* spores and released metabolites, and absence of symptoms) could represent a pre-infection/early stage of infection. The observed Cu speciation changes correlating with this inoculation thus suggest that the fungal metabolites and applied together on plant leaves changed the chemical environment of the leaf surface, leading to Cu formulation transformations, as discussed below.

**Fig. 4:**
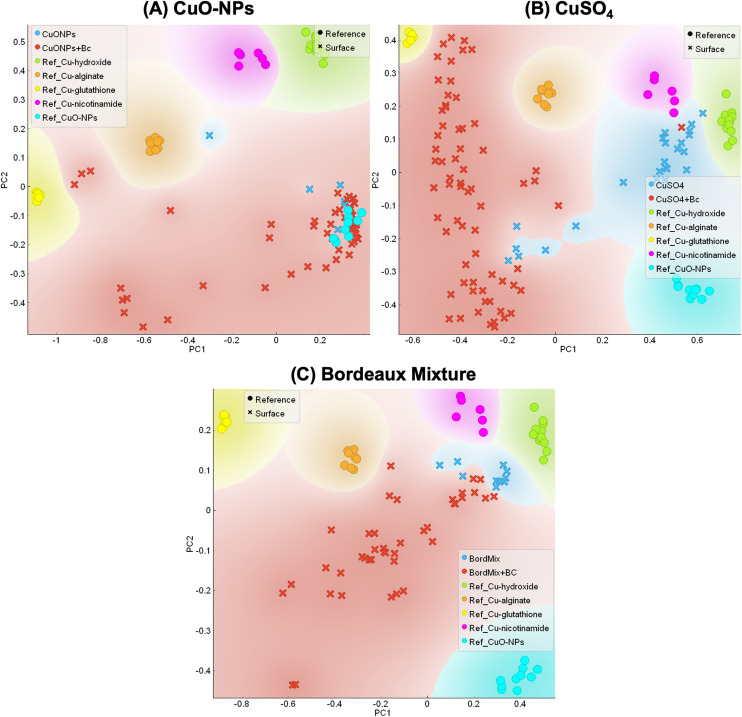
PCA analysis representing XANES results for (A) CuO-NPs, (B) CuSO_4_, and (C) Bordeaux mixture, for control (non-inoculated, in blue) and *B. cinerea*-inoculated samples (red). Samples are compared to the following references: Cu–hydroxide (green), Cu–alginate (Cu–carboxyl, orange), Cu–thiol (Cu–glutathione, yellow), Cu–nicotinamide (Cu–amine, pink) and CuO (CuO-NPs, turquoise). Circles indicate Cu references and exes indicate samples' POIs.

For all treatments, μ-XANES results show that leaf inoculation caused Cu dissolution, complexation and reduction (Fig. 4), as a fraction of Cu was found associated with thiol, hydroxyl and carboxyl groups. These Cu speciation changes are likely due to biomolecule releases (*e.g.* proteins, enzymes, metalloproteins) by the fungi. It has been previously shown that *B. cinerea* produces oxalic acid, catechol-type siderophores, and metallothionein (cysteine rich),^30^ as well as glutathione peroxidase^63^ both *in planta* and *in vitro*. It is hypothesized that oxalic acid, when dissociating to oxalate and protons, lowered the leaf surface's pH. This pH change is required for the pathogen colonization^36^ both *in vitro* and *in planta*^30,36^ and could have triggered brochantite and CuO-NPs dissolution. Moreover, it can be hypothesized that compounds produced by the fungus and/or the plant provided ligands for ionic Cu binding, leading to a change in Cu speciation that was either internalized by plant/fungal cells or chelated at the leaf surface. These compounds can be: (i) fungal compounds produced on the leaf surface or *in vitro* that were added to the leaf surface along with the inoculation and/or (ii) plant compounds, produced in response to the stress caused by the presence of the fungus and/or the Cu applied to the leaves.^36,37,63^

It is worth noting that in all samples, there is a group of POIs that are not associated with any of the Cu references used (Fig. 4 and S11). This is even clearer in the Bordeaux mixture, compared to the other Cu formulations. In particular, this speciation suggests Cu reduction, and the literature suggests this XANES profile can be a complexation of Cu(i) with thiol.^64^ This type of Cu(i)–thiol bounding is typical in slightly acidic and reducing intracellular environment^64^ and it is likely a result of electron transfer when thiol binds to Cu(ii), reducing it to Cu(i). This speciation change could also be related to responses of the leaf phyllosphere due to the presence of *B. cinerea*. It is known that grapevines' leaf microbiome can induce tolerance/resistance to foliar pathogens,^65^ therefore, *B. cinerea* inoculation could have triggered the production of metabolites by the phyllosphere in order to respond to this biotic stress.^66^ Nonetheless, the production of compounds with Cu–thiol speciation by the plant, in response of the stress induced by both the Cu and the pathogen presence cannot be excluded.^67,68^

### Copper persistence in the exposed tissues

**Fig. 5:**
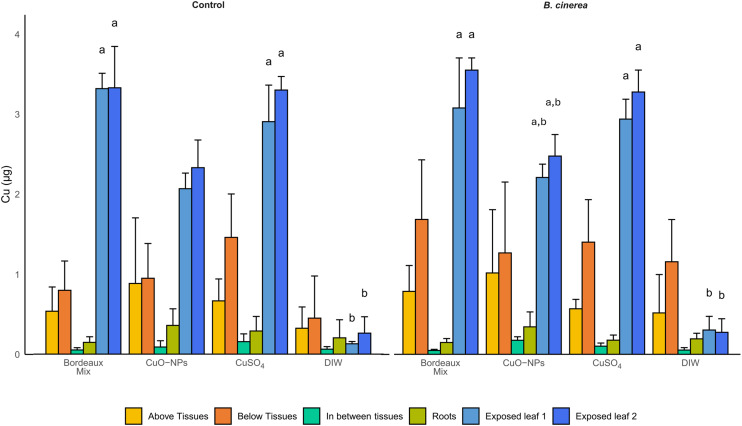
Cu quantification in exposed tissues (exposed leaves, blue) and non-exposed tissues, namely, the tissues above the exposed leaves (above tissues, yellow), tissues between the two exposed leaves (in between tissues, aqua), tissues below the exposed tissues (below tissues, orange) and roots (green), for all formulations, with inoculation of *B. cinerea* spores and without (control). Error bars represent the standard deviation among the replicates. Letters indicate statistically significant differences between Cu formulations, for the exposed leaves only (Kruskal–Wallis followed by pairwise Dunn test with Bonferroni *p*-value correction were performed). There are no differences between Cu formulations, for either the control or the inoculated sample in non-exposed tissues (above, below, and in between tissues and roots). There are no statistically significant differences between the control and inoculated sample for the same Cu formulations.

The translocation of Cu to non-exposed tissues after uptake was assessed by analyzing the roots, tissues below, in between and above the exposed leaves (Fig. 5). The results of the Cu concentration measured in the initially prepared solutions/suspensions as well as the theoretically applied Cu mass are presented in Table S3. Results regarding the recovered Cu in the plants *vs.* theoretical initially applied Cu are presented in Table S4.

The Cu initially applied, namely 3.3 μg per leaf (6.6 μg per plant), was recovered in the collected tissues (Table S4), mainly in the exposed leaves. The total recovered Cu per plant for CuO-NPs-exposed plants was 90 ± 17% and 85 ± 21%, for the control and inoculated plants, respectively. In CuSO_4_-exposed plants, Cu recovery was 116 ± 7% and 94 ± 19%, for the control and inoculated plants, respectively. For plants exposed to Bordeaux mixture, in control plants 108 ± 6% of Cu was recovered and in the inoculated plants, the Cu recovered was 109 ± 25% (see Table S4 for further details).

Most of the Cu applied to plant leaves were recovered on/in the exposed leaves (Fig. 5), suggesting limited-to-no Cu translocation to non-exposed tissues after uptake. This is valid for all the formulations, with and without the presence of *B. cinerea*. These results further suggest that even though Cu transformations occurred after foliar application, there is limited Cu uptake and/or low Cu mobility within the plant system, for the 9 days of exposure. Aside from experiments performed in grapevines,^15^ absence of Cu translocation following CuSO_4_ and CuO-NPs foliar application has been previously observed in lettuce.^13^ Overall, results indicate that the plant responses to the *B. cinerea*'s presence and/or the complexation of Cu by metabolites did not result in an increase in Cu mobility within the plant, suggesting that Cu homeostasis was well regulated *in planta*, even in the presence of this phytopathogen.

Persistence of Cu at the exposed leaves suggests potential for more effective plant protection in grapevines above-ground tissues due to reduced translocation.^69,70^ Since Cu uptake is limited, the applied plant protectant may remain at the surface and/or in the first layers of the leaf and likely still protecting the plant from pathogens. This in turn could potentially lead to a reduction in the application frequency and intensity of the fungicides needed to protect the plant tissue.

## Conclusions

The present work demonstrated that the initial Cu formulation impacted both Cu distribution and transformations after foliar application, in non-inoculated leaves. The Cu signal in the μ-XRF maps suggests Bordeaux mixture remained largely undissolved/untransformed on the surface of the leaf, due to the higher crystal size (>2 μm) of brochantite mineral formed in the Bordeaux mixture. A fraction of Cu from CuO-NPs seemed to be taken up and vasculature-loaded, but most of the Cu remained untransformed at the leaf surface during the 9 days of the experiment. It is likely that CuO-NPs were taken up, dissolved and complexed after entering the leaf.^15^

While Cu in non-inoculated leaves mainly remained untransformed, in inoculated leaves, Cu was found transformed for all Cu formulations. The inoculation of *B. cinerea* could mimic a pre-infection scenario, that led to the dissolution, complexation and partial reduction of Cu by thiol groups at the leaf surface. *B. cinerea*-mediated transformation was likely due to several processes, including the production of organic acids (*e.g.* oxalic acid) *in vitro*, usually associated with a decrease of environmental pH leading to Cu (nano)formulations' dissolution. Complexation likely followed this dissolution due to the presence of catechol siderophores and/or thiol-rich enzyme and proteins, all involved in the mitigation of plant-produced ROS in response to pathogen infection^63^ and in the complexation of metal elements.

The observed biotransformation had no observable impact on the translocation of Cu to other plant tissues. This could be considered as a positive result, as even in the presence of a pathogen, the applied Cu formulations remained on/in the initially applied tissues. These results further suggest that CuO-NPs have the potential for Cu^2+^ release upon trigger by fungal metabolites. CuO-NPs can persist in their nanoform with enhanced adhesion to the leaf surface, until exposure to a pathogen and/or its metabolites. Upon this contact, dissolution of the CuO-NPs might occur, resulting in the release of their primary antifungal Cu species. Additionally, no Cu translocation to non-exposed tissues along with our previous work highlighting no detection of CuO in nanoforms in the petiole,^15^ suggests that the foliar-applied CuO-NPs did not persist in the nanoform within the plant system. Concerns of the impact of NPs in both human health and environmental safety have been a debate for years,^27,71–74^ and this observation suggests that there may be a low risk of NPs intake by human consumption due to translocation within the plant system since it is unlikely that CuO-NPs will reach the edible part of grapevines, namely the grapes, in the nanoform. Nonetheless, application of Cu-based formulations in grapevines occurs whenever protection from disease outbreaks are needed,^69,70^ and this can happen upon fruiting season, therefore, ingestion due to application in fruits still needs to be considered along with the application strategy. Notably, these highlight the importance of complex *in planta* studies.

Additional experiments are needed to understand if these results, obtained in controlled conditions, namely germinated grapevines potted in sand to minimize the Cu background, may hold true in a realistic scenario with grafted grapevines. For instance, leaf surface properties, namely stomatal and trichome density, or cuticle thickness, will likely change from seedlings to mature grapevines^75^ which could affect the interaction among the CuO-NPs, grapevines and the pathogen. Consequently, leaf adhesion and translocation within the plant system may differ from younger plants. Additionally, experiments performed in vineyards will allow long-term experiments^57^ to better capture the life cycle of these products, their impacts, as well as assessing the cost of Cu nanoformulations usage as preventive fungicides. Research suggests that foliar delivery of nanoformulations for plant protection could be economically valuable comparing to bulk counterparts, due to targeted delivery, possibly decreasing the application dose,^76^ while increasing their efficacy. Current evidence indicates that some classes of products are considered to be ready for validation/commercialization.^77^ Yet, to date, these calculations are based on results performed on highly control conditions and need to be adjusted to data collected under more realistic scenarios, including a thorough life-cycle analysis of the products.

A more complex/realistic scenario will allow to address not only the environmental fate of these nano-based fungicides to properly evaluate their benefits-over-risks^27,74^ but also the fungicide effect of CuO-NPs compared to conventional fungicides (*e.g.* Bordeaux mixture). Indeed, future studies with experimental setups that promote and/or monitor disease development are essential to elucidate if the observed biotransformations could have an impact on the fungicidal activity of Cu formulations. Additional identification/characterization of fungal metabolites produced *in planta* during several stages of disease development may elucidate the compounds playing a role on the Cu-based formulations' transformations. Time-series follow-ups should be considered to capture and disentangle Cu transformation kinetics in the longer term. Simultaneously, plant defense responses should also be monitored since the produced compounds (*e.g.* SOD, CAT, PPO enzymes) may also modulate Cu speciation changes and subsequent Cu formulation efficiency. Additional experiments using other grapevine fungal pathogens, especially ones tackled with Cu-based fungicides, would give insights regarding the strategies used by foliar pathogens to cope with the presence of Cu, further understanding the modulation of Cu fungicides' reactivity. Similarly, as the results presented here showed that the presence of *B. cinerea* alters Cu speciation, the role of the phyllosphere in general elemental fate seems to be important to address in the future, as well as the impact of Cu-based formulations on the leaf microbiota itself.^62,78,79^

These findings provide valuable insights regarding the pathogen modulation of plant-applied Cu-based formulations. This knowledge not only helps to understand the NP-plant interactions but may also help to improve the Cu dose currently applied of conventional fungicides while addressing the conditions where CuO-NPs can present plant protection properties. Consequently, this knowledge may be helpful for designing safer and efficient nano-based fungicides for the near future. More broadly, this work also demonstrates the mutual interaction occurring at the interfaces of (Cu) formulations deposition, the phyllosphere, and the plant, due to biological and chemical changes. It highlights that understanding these interplays is needed to better design plant protection products responding to biological triggers but also understand how foliar treatment can shape and impact the phyllosphere.

## Conflicts of interest

The authors declare no known competing or personal interests that could have influenced the work reported in this paper.

## Supplementary Material

EN-013-D5EN01102G-s001

## Data Availability

The authors declare that the data supporting this study are available either in the paper or in the associated SI. The dataset generated at the ESRF is available at: https://doi.esrf.fr/10.15151/ESRF-ES-1568128044. The raw data files analyzed in this study will be provided by the corresponding author upon reasonable request. Supplementary information (SI) is available. See DOI: https://doi.org/10.1039/d5en01102g.
