# Supplementary material for: Evidence of copper (nano)formulation biotransformations triggered by Botrytis cinerea on grapevine leaves for targeted plant protection
Source: Environ Sci Nano. 2026 Feb 24;13(3):1485–98. doi: 10.1039/d5en01102g (PMC12930394; doi:10.1039/d5en01102g)
Supplement: EN-013-D5EN01102G-s001 [file EN-013-D5EN01102G-s001.pdf]

Supplementary Information (SI) for Environmental Science: Nano.
This journal is © The Royal Society of Chemistry 2026

Supporting information

for

Evidence of copper (nano)formulation biotransformations triggered by Botrytis cinerea on grapevine leaves for targeted plant protection

Diana Salvador[1], Sónia Rodrigues[2], Artur Alves[1], Cátia Fidalgo[1], Sandra Rodrigues[2], Hiram Castillo-Michel[4] & Astrid Avellan[5]

1. Department of Biology and Centre for Environmental and Marine Studies (CESAM), Universidade de Aveiro, Portugal
2. Department of Environment and Planning and Centre for Environmental and Marine Studies (CESAM), Universidade de Aveiro, Portugal
4. European Radiation Synchrotron Facilities, Grenoble, France
5. Géosciences Environnement Toulouse (GET), CNRS, UMR 5563, UT3, IRD, CNES, OMP, France

1. Methods

Dissolution tests

Tests to evaluate the Bordeaux Mixture dissolution were performed at 1 mg/L in distilled water (DIW) under agitation for 7 days. Aliquots were collected at 0, 3 and 7 days. At each collection, two aliquots of 2 mL were collected: one for total Cu analysis and a second, for analysis of the dissolved Cu. The total Cu aliquot was immediately acidified with HNO_3 for ICP-MS analysis while the second aliquot was centrifuged for 30 minutes at 16 000 g, ¼ of the total volume was collected and placed in a new tube, diluted and acidified for ICP-MS analysis. The dissolution % was calculated by the following formula:

$$Dissolution\,\% = \frac{m\ dissolved\ Cu\ (mg)}{m\ total\ Cu\ (mg)} \times 100$$

Sand acidic washing

Sand was washed with DIW until the running water was clear. The sand was then completely immersed in a solution of nitric acid at 3% and left overnight. The next day, the acid was removed, and the sand was washed five times with DIW, or until the pH of the damped sand was approximately 4-5. The sand was washed twice with Hoagland Solution (without Cu) to increase the pH to 5-6 and then washed once with DIW. The washed sand was then placed at 200ºC overnight. After this, the sand was left to cool down and washed once with DIW before potting the plants. Cu concentration in the sand was measured by ICP-MS and each pot contained approximately $4.91x10^{-5} \pm 1{,}27\ x10^{-5}$ µg of Cu.

Hoagland Solution

Table S1. Contribution of each macro and micronutrient in the ¼ strength modified Hoagland solution prepared. This nutrient solution was prepared without Cu to limit the Cu supply. Grapevines were not Cu deprived as the substrate where they were grown had Cu in its composition.

**Macronutrients**	**Concentration (mM)**
KNO_3	1.29
$Ca(NO_3)•4H_2O$	1.20
$MgSO_4•7H_2O$	0.50
KH_2PO_4	0.25
**Micronutrients**	
NaFe(III)-EDTA	5.00×10^{-3}
H_3BO_3	11.56×10^{-3}
$Na_2MoO_4•2H_2O$	0.12×10^{-3}
$ZnSO_4$	0.19×10^{-3}
$MnCl_2$	2.29×10^{-3}

List of Cu standards used in XANES analysis

CuO-NPs, $CuSO_4$, $Cu(OH)_2$, $CuCl_2$, $Cu_3(PO_4)_2$, and Cu-oxalate were purchased and prepared for XANES analysis at 1 g of Cu/L prior to the beam time analysis. Cu-alginate, Cu-citrate, Cu-cysteine, Cu-glutathione, and Cu-nicotinamide were synthesized in the laboratory based on adaptations of previously published experimental protocols. Detailed description of each Cu standard synthesis is provided in previous work (1).

Table S2. List of Cu standards used in XANES. (¥) represents the references used for both the analysis and representation of the experimental data.

**Standard**	**Type of bond**
Cu-alginate ¥	**Cu-O-R**
Cu-citrate	Cu-O-R
Cu-cysteine	Cu-S-R
Cu-glutathione ¥	**Cu-S-R**
Cu-chloride ($Cu(Cl)_2$)	Cu-Cl
Cu-nicotinamide ¥	**Cu-N**
CuO-NPs ¥	**Cu-O**
Cu-hydroxide ($Cu(OH)_2$) ¥	**Cu-OH**
Cu-oxalate	Cu-O-R
Cu-phosphate ($Cu_3(PO_4)_2$)	Cu-O-P
Cu-sulfate ($CuSO_4$) ¥	**Cu-O-S**

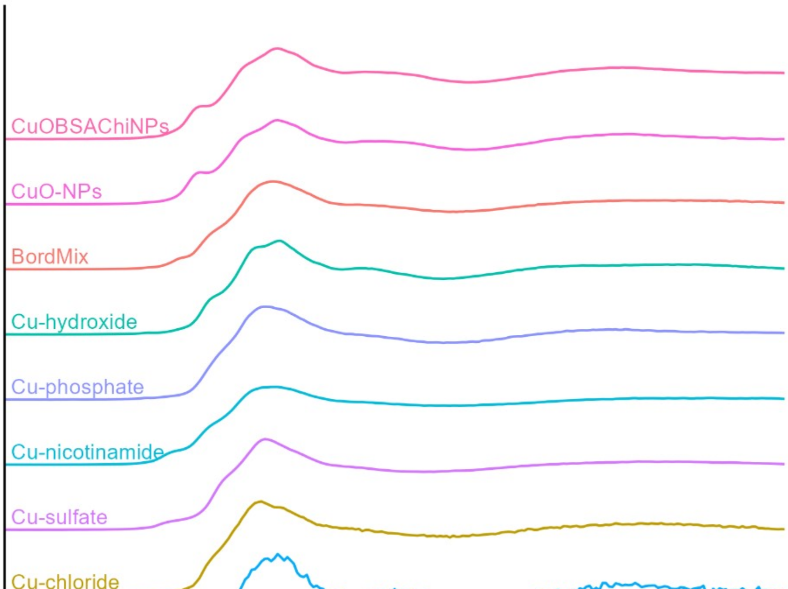

Figure S1 – XANES standards analyzed at the beamline ID-21.

2. Results

<u>Cu formulations speciation</u>

Bordeaux Mixture, $CuSO_4$ and CuO-NPs, at 1 g of Cu/L were loaded on the beamline ID-21 at the European Synchrotron Radiation Facility (ESRF), to infer about their Cu speciation. The figure below (Fig. S2A) shows all the references used at the beamline session. Details on the PCA analysis performed are presented in section 2.4.2. of the main article. Fig. S2B shows that Cu signal in the Bordeaux Mixture is not the same as $Cu(OH)_2$. See Fig. S3 for details on the Linear Combination Fit (LCF) analysis for Bordeaux Mixture.

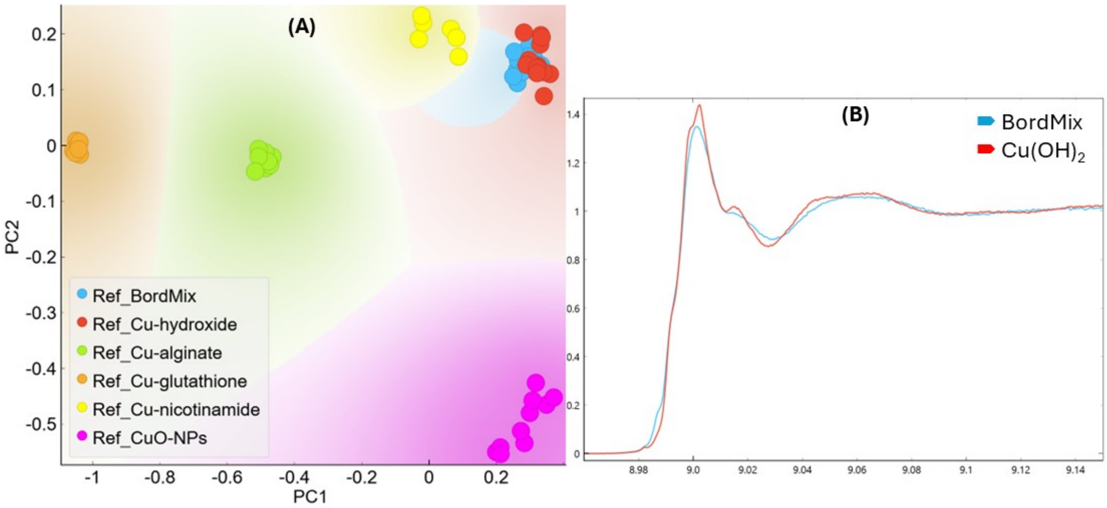

Figure S2 – (A) PCA analysis of µ-XANES spectra of the several Cu compounds used as standards for the speciation analysis and (B) spectra of $Cu(OH)_2$ (in red) and Bordeaux Mixture (blue).

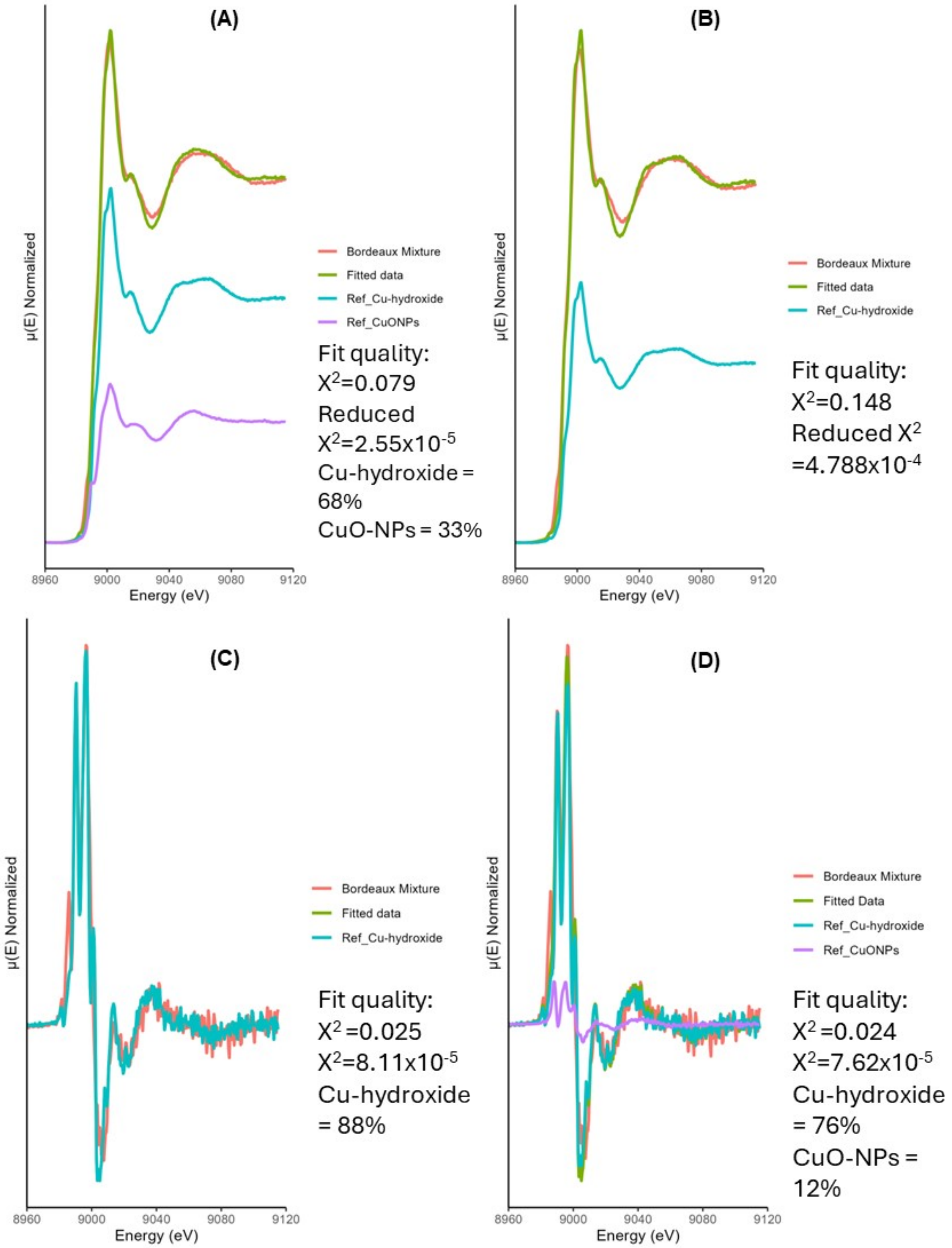

Figure S3 – Different linear combination fits (LCF) of Bordeaux Mixture to elucidate on the decision-making process for the best fit.

<u>Cu formulations dissolution</u>

Dissolution assays were performed for Bordeaux Mixture (see ‘Dissolution tests’ in the Methods section of this file for further information). Results of Bordeaux Mixture and CuO-NPs are presented in the figure below. CuO-NPs data used for comparison were performed in a previous

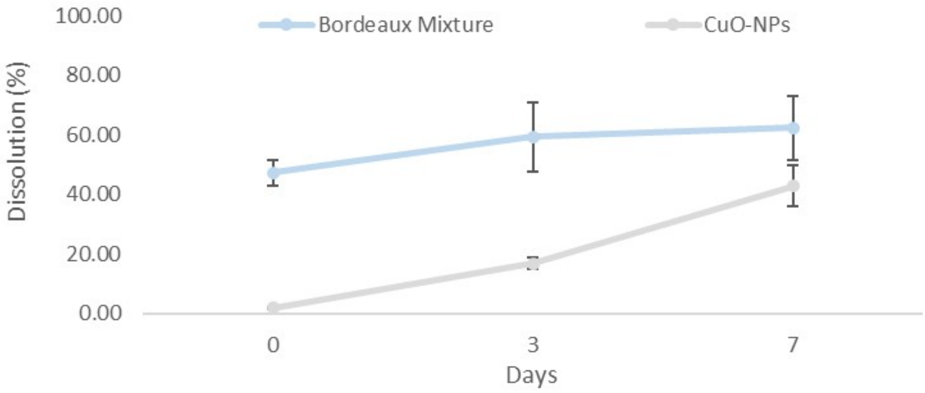

Figure S4 – Dissolution tests of Bordeaux Mixture in distilled water (DIW), for 0, 3 and 7 days, in comparison to CuO-NPs. CuO-NPs data were previously published in (1).

study (1).

<u>μ-XRF maps of the leaf surface 9 days after Cu exposure, highlighting the leaf vasculature</u>

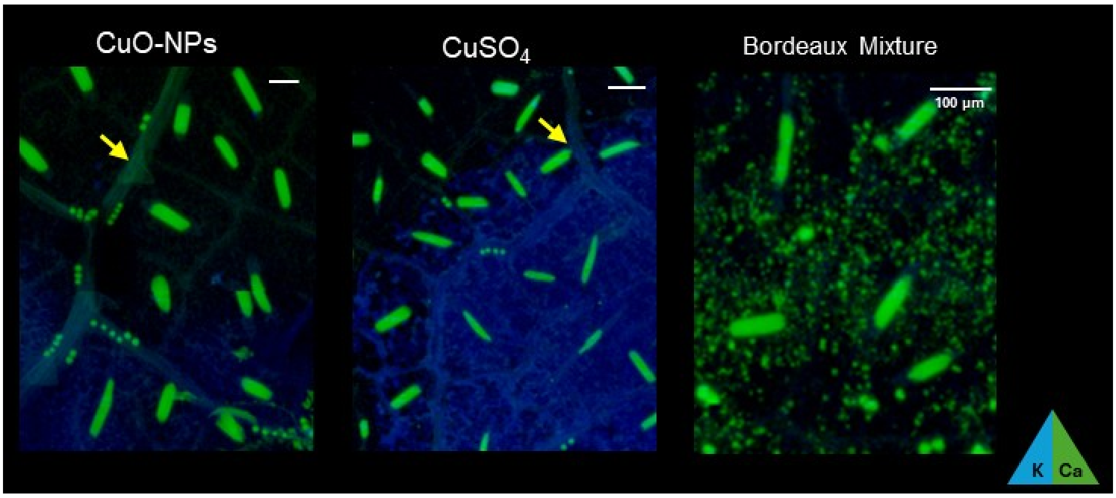

Figure S5 - μ-XRF maps showing the leaf surface without the Cu signal, to highlight the plant features, namely the vasculature (yellow arrows).

POIs of surface detected Cu in $CuSO_4$-exposed leaves: highlighting the POIs not associated with a reference

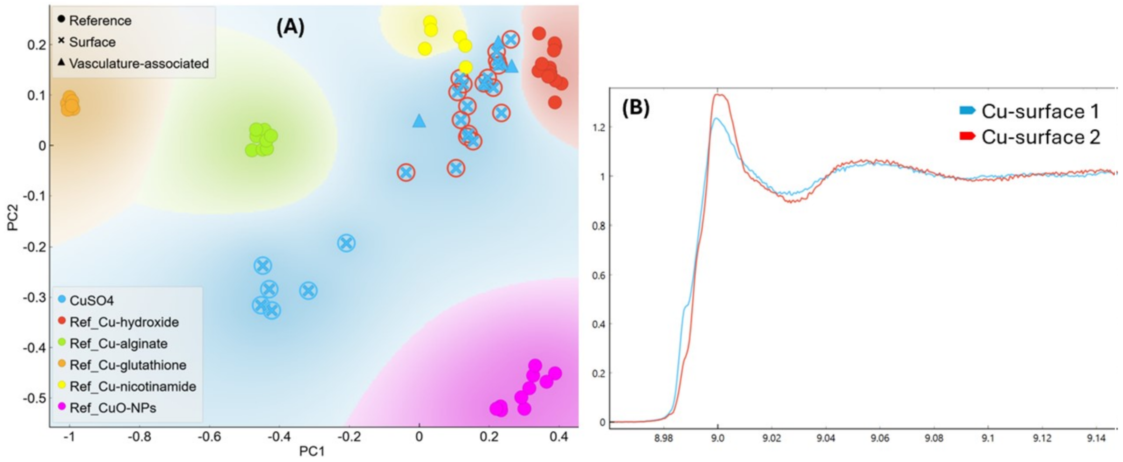

Figure S6 – (A) PCA representing the XANES analysis of $CuSO_4$-exposed leaves, highlighting the surface-associated POIs not associated with any of the selected references (blue), comparing to the remaining surface POIs (red) and (B) the respective spectra.

Cu speciation of all foliarly applied formulations

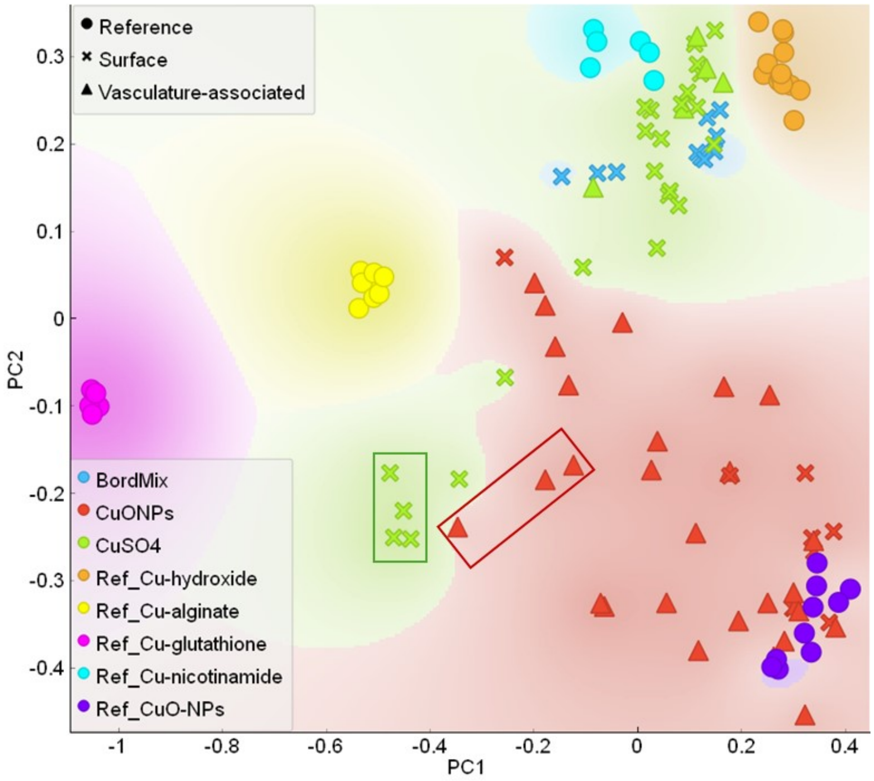

Figure S7 – PCA representing the XANES analysis of all the Cu-exposed leaves. Here, it is possible to observe the similar tendency between the surface POIs of the $CuSO_4$-exposed leaves (green) and the vasculature POIs of CuO-NPs-exposed leaves (red).

Spores of *Botrytis cinerea* at the leaf surface, 9 days after Cu exposure and 6 days after leaf inoculation: µ-XRF and SEM micrographs

1. µ-XRF maps:

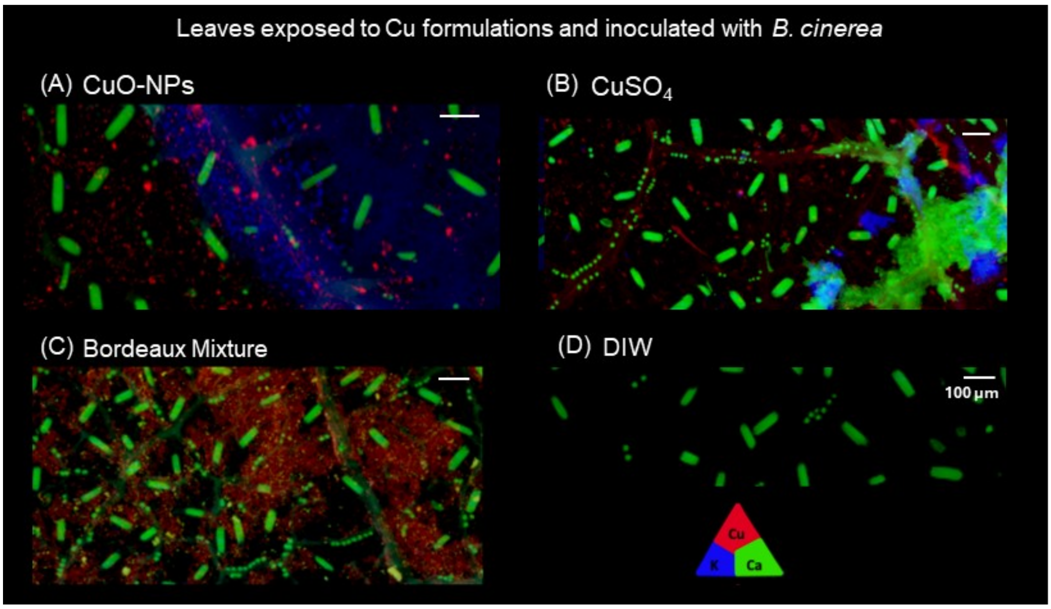

Figure S8 - µ-XRF maps of grapevines exposed to (A) CuO-NPs, (B) $CuSO_4$, (C) Bordeaux Mixture and (D) DIW control, and later inoculated with *Botrytis cinerea* spores. These maps were collected after 9 days of Cu exposure and 6 days after leaf inoculation. The triangle represents the color code used: Potassium (K) is represented in blue, calcium (Ca) in green and copper (Cu) in red. White bar represents 100 µm.

2. SEM micrographs:

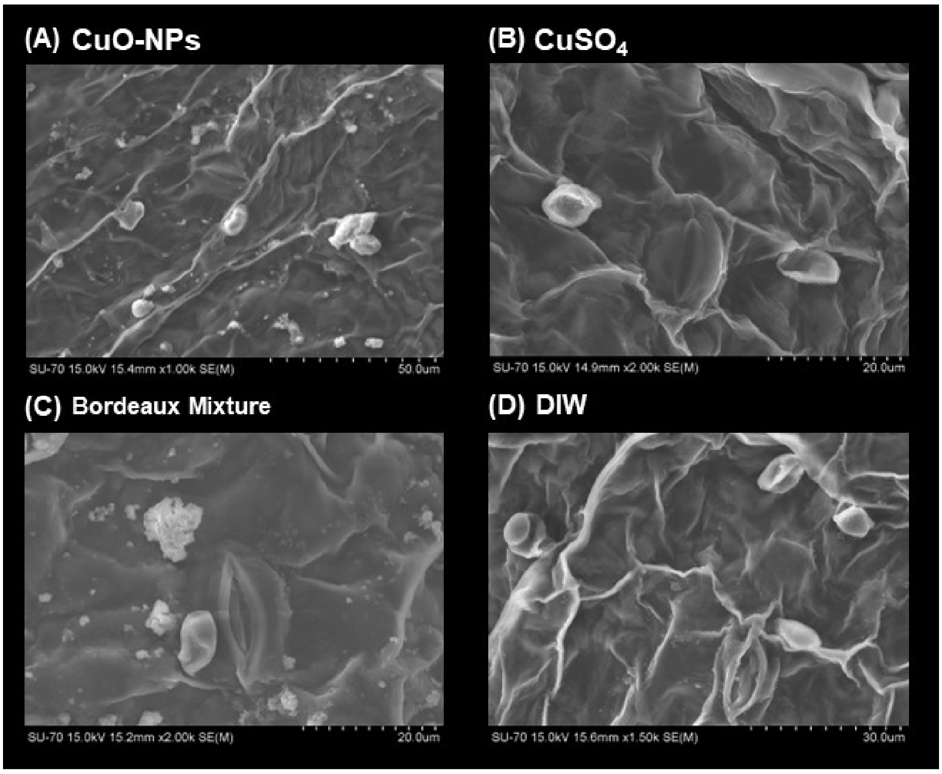

Figure S9 – SEM micrographs of *Botrytis cinerea* spores at the surface of leaves exposed to: (A) CuO-NPs, (B) $CuSO_4$, (C) Bordeaux Mixture, and (D) DIW. Images were captured 9 days after Cu exposure and 6 days after leaf inoculation.

3. µ-XRF map of *Botrytis cinerea* spores:

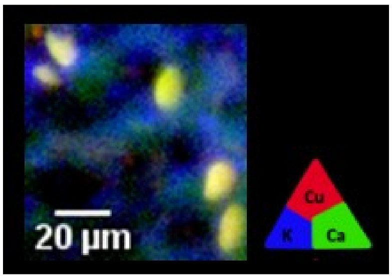

Figure S10 – μ-XRF maps of *Botrytis cinerea* spores on top the grapevines leaves. The elements represented in the map are: copper (red), potassium (blue), and chloride (green).

Spectra of POIs with unknown speciation of leaves exposed to $CuSO_4$, CuO-NPs and Bordeaux Mixture and inoculated with *B. cinerea*

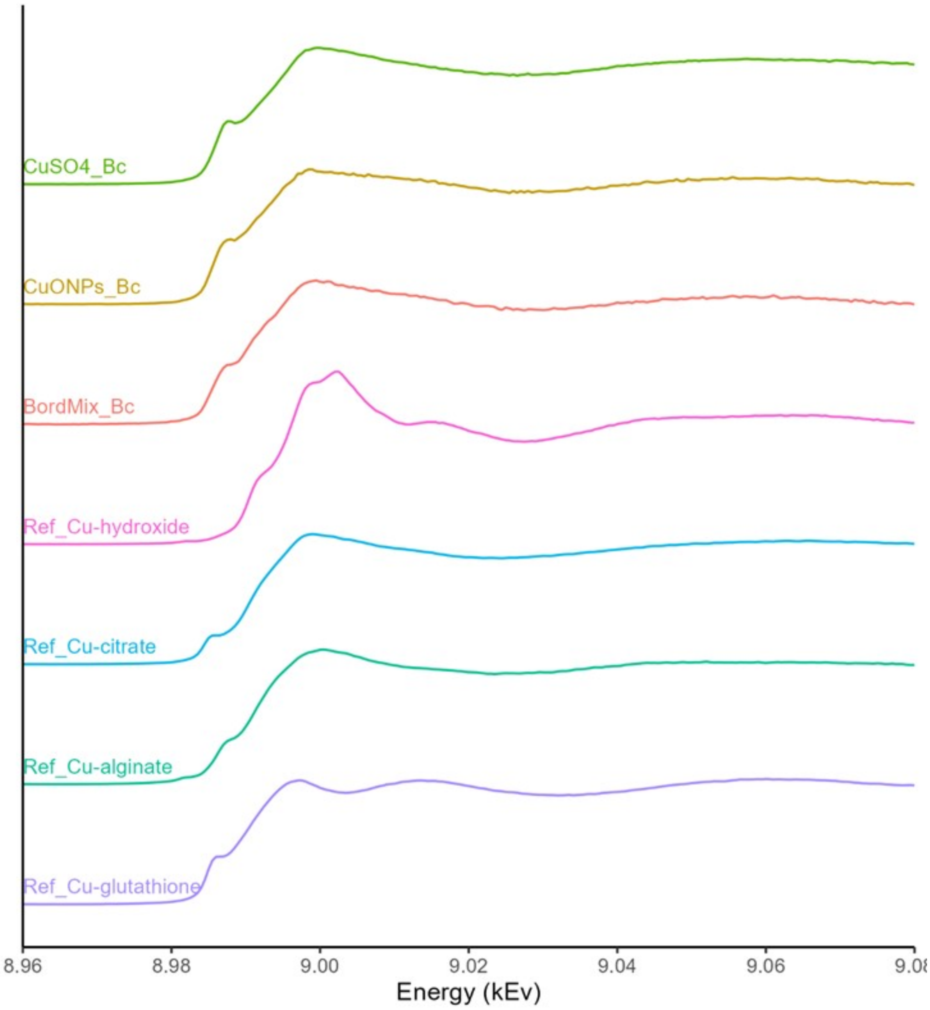

Figure S11 – XANES spectra of POIs with unknown speciation of leaves exposed to the Cu formulations, namely, $CuSO_4$, CuO-NPs and Bordeaux Mixture, later inoculated with spores of *Botrytis cinerea*. The samples are compared to the following references: $Cu(OH)_2$ (Cu hydroxide), Cu-citrate and Cu-alginate (Cu-carboxyl) and Cu-glutathione (Cu-thiol).

Mass of Cu added to the plants

Table S3. Initially applied Cu mass (µg) considering the concentration measured (by ICP-MS) in the initial solutions/suspensions prepared.

Cu formulation	Initial solution/suspension (g/L)	Applied volume per plant (µL)	Theoretical initially applied mass (µg)
CuO-NPs	0.0244	240	5.850
CuSO4	0.0264		6.346
Bordeaux Mixture	0.0261		6.265
DIW	1.704×10^{-5}		0.004

Total Cu quantified per plant

Table S4. Cu mass (µg) recovered per plant. The result includes the sum of the Cu quantified in the exposed leaves, the tissues above, below, and between the exposed leaves, and the roots.

Cu formulation	Average total Cu in the plant (µg)	Standard Deviation	Theoretical initially applied mass (µg)	Recovered Cu (%)
CuO-NPs	6.674	0.906	5.850	90 ± 17
CuO-NPs + *B. cinerea*	7.492	0.577		85 ± 21
$CuSO_4$	8.774	0.339	6.346	116 ± 7
$CuSO_4$ + *B. cinerea*	8.471	0.624		94 ± 19
Bordeaux Mixture	8.177	0.213	6.265	108 ± 6
Bordeaux Mixture + *B. cinerea*	9.303	1.200		109 ± 25
DIW	1.431	0.301	-	-
DIW + *B. cinerea*	2.503	1.030		-

3. References

1. Salvador D, Miranda M, Rodrigues S, Castillo-Michel H, Fidalgo C, Alves A, et al. Elucidating nano-Cu interactions in grapevine leaves: formulation-dependent foliar affinity, uptake, and leaf persistence over time. Environ Sci Nano [Internet]. 2025; Available from: http://dx.doi.org/10.1039/D5EN00322A
